# Genome-Wide Dynamic Evaluation of the UV-Induced DNA Damage Response

**DOI:** 10.1534/g3.120.401417

**Published:** 2020-07-30

**Authors:** Erica Silva, Manuel Michaca, Brenton Munson, Gordon J. Bean, Philipp A. Jaeger, Katherine Licon, Elizabeth A. Winzeler, Trey Ideker

**Affiliations:** * Department of Medicine, University of California, San Diego, La Jolla, California; † Department of Bioengineering, University of California, San Diego, La Jolla, California; ‡ Bioinformatics and Systems Biology Program, University of California, San Diego, La Jolla, California; § Division of Host-Microbe Systems & Therapeutics, Department of Pediatrics, University of California, San Diego, California

**Keywords:** ultraviolet radiation response, DNA damage response, high-throughput screen

## Abstract

Genetic screens in *Saccharomyces cerevisiae* have allowed for the identification of many genes as sensors or effectors of DNA damage, typically by comparing the fitness of genetic mutants in the presence or absence of DNA-damaging treatments. However, these static screens overlook the dynamic nature of DNA damage response pathways, missing time-dependent or transient effects. Here, we examine gene dependencies in the dynamic response to ultraviolet radiation-induced DNA damage by integrating ultra-high-density arrays of 6144 diploid gene deletion mutants with high-frequency time-lapse imaging. We identify 494 ultraviolet radiation response genes which, in addition to recovering molecular pathways and protein complexes previously annotated to DNA damage repair, include components of the CCR4-NOT complex, tRNA wobble modification, autophagy, and, most unexpectedly, 153 nuclear-encoded mitochondrial genes. Notably, mitochondria-deficient strains present time-dependent *insensitivity* to ultraviolet radiation, posing impaired mitochondrial function as a protective factor in the ultraviolet radiation response.

Genome-wide screening techniques in the model organism *Saccharomyces cerevisiae* have permitted extensive functional annotation of nearly every gene (Winzeler *et al.* 1999; Schuldiner *et al.* 2005; Breslow *et al.* 2008; Baryshnikova *et al.* 2010; Douglas *et al.* 2012; Kofoed *et al.* 2015). In such screens, the relative contribution of each gene is often determined according to the fitness of the corresponding gene knockout strain, as inferred from macroscopic phenotypes, such as colony size (Costanzo *et al.* 2010; Baryshnikova *et al.* 2010; Kuzmin *et al.* 2014; Bean *et al.* 2014) or relative strain abundances (Winzeler *et al.* 1999; Giaever *et al.* 2002; Breslow *et al.* 2008; Schlecht *et al.* 2017). However, biological processes are dynamic (Celaj *et al.* 2017); isolated snapshots may not adequately describe their full complexity (Bandyopadhyay *et al.* 2010). Furthermore, genetic perturbations may not always result in notable changes in the observed colony fitness, as defects may be small (Thatcher *et al.* 1998; Baryshnikova *et al.* 2010; Styles *et al.* 2016), transient or context-dependent (Styles *et al.* 2016).

To address these limitations, additional assays have been directed at the capture of dynamic responses. For example, high-throughput fluorescence imaging studies can characterize microscopic phenotypes such as dynamic protein localizations and abundances (Dénervaud *et al.* 2013; Kraus *et al.* 2017). Although limited in scalability, liquid micro-culture assays, in which the growth curves of mutant strains are analyzed, permit characterization of dynamic growth responses as well as identification of marginal fitness phenotypes (Warringer *et al.* 2003; Toussaint and Conconi 2006). Recent efforts have been made to improve scalability of growth curve analysis by leveraging existing genetic mutant colony array technology (Hartman and Tippery 2004; Shah *et al.* 2007; Banks *et al.* 2012; Zackrisson *et al.* 2016; Barton *et al.* 2018).

The DNA damage response (DDR) is a collection of complex and dynamic mechanisms that ensures detection and repair of DNA damage as well as coordination of repair with other cellular physiological processes such as cell cycle arrest and damage tolerance. Ultraviolet radiation (UVR) is a ubiquitous environmental source of DNA damage, mostly in the form of UV-A (320-400nm) or UV-B (280-320nm) waves. UV-C waves (200-280nm) are largely filtered by the atmosphere (Matsumura and Ananthaswamy 2004), but, being most efficient in DNA-damaging ability (Ravanat *et al.* 2001), are routinely used in research. UVR primarily causes the formation of helix-distorting cyclobutane pyrimidine dimers (CPDs) and 4-6-photoproducts (4-6PPs), which are repaired by the nucleotide excision repair (NER) machinery. UVR also induces lower levels of oxidative DNA damage, single-strand breaks, and protein-DNA crosslinks (de Gruijl *et al.* 2001; Cadet and Wagner 2013), which are repaired by base excision repair and other machinery (Prakash and Prakash 2000; Sinha and Häder 2002; Schärer 2013). The DDR is linked to many other cellular processes, such as transcription, replication, ubiquitination, and the cell cycle, highlighting the dynamic, interconnected nature of this process (Prakash and Prakash 2000; Srivas *et al.* 2013).

Here, we combine classical fitness measurements (*i.e.*, colony fitness, CF) with a dynamic fitness evaluation technique, Genome-wide Evaluation Of Dynamic Events (GEODE), to examine the response of *S. cerevisiae* to UV-C radiation. In addition to established DNA repair genes, we find components of the CCR4-NOT complex, autophagy, and tRNA wobble uridine modification. We also unexpectedly find that many strains deficient in genes with mitochondrial functions are insensitive to UVR-induced DNA damage, posing impaired mitochondria as a protective factor in the UVR response.

## Materials and Methods

### Yeast strain identification

We chose to screen the diploid homozygous knockout yeast library (ATCC, GSA-7). To validate all strain identities, we designed a sequencing strategy by which to identify strains based on the unique barcodes incorporated into the Yeast Knockout Library. Primers (Table S1) capable of amplifying the UPTAG region (strain-specific barcode) were designed such that the forward primer contained a well-specific barcode. Combining this well-specific barcode with the amplified UPTAG allowed us to uniquely identify strains and their plate locations via pooled sequencing. The diploid library was found to contain 4467 unique strains (See Figure S1, File_S1 for Supplementary Methods).

### Library maintenance and screening protocol

Using a Singer pinning robot (Rotor 100, Singer Instruments), the library was up-scaled from 96 to 384-format. A liquid-handling robot (Freedom Evo 200, Tecan) was used to re-array the library such that each edge colony also appeared inside the plate. The yeast array was maintained on agar + YPAD in 1536 format under G418 selection at 4C (for storage) or room temperature (for growth). The evening prior to screening, 1536 plates were replicated onto 2% carrageenan plates, prepared as previously described (Jaeger *et al.* 2015) containing synthetic complete media (without G418) and grown overnight at room temperature. To screen, the collection was upscaled to 6144-density onto pre-warmed 2% carrageenan plates which were then placed facedown (without lids) inside an imaging light-box on a sanded, black acrylic surface. Plates were imaged with a Nikon D800e camera, fitted with an AF Micro Nikon 60mm lens, using Camera Control Pro 2 Software (Nikon). Grayscale images were taken at five-minute intervals and stored as TIFF images. For UVR treatment, plates were taken from the setup immediately after image #48 (4 hr), placed, face-up without lid, into a UV cross-linker (Hoeffer UVC500-115V) and treated with 15 × 10^3^ µJ/m^2^ UV-C. They were immediately placed back into the imaging station before image #49 was taken at the next five-minute interval (*i.e.*, no images were missed due to UVR treatment). Imaging was continued up to 48 hr. The experimental setup was repeated nine times, resulting in 18 plates per condition. In further analysis, three of 18 plates were removed from analysis due to insufficient imaging time.

### Image analysis

Images were processed using MATLAB Colony Analyzer Toolkit V2, which we make available. Image crops were defined manually for each plate before and after UV treatment; colony grid placements were manually defined for each plate (images 48, 49, 300) using *ManualGrid()* and were reused for other images. Images were smoothed using MATLAB’s *imdiffusefilt()* with default settings. Colony borders were established with *HalfModeMax()*. Colony area and colony intensity (*i.e.*, the sum intensity of the pixels constituting a colony) were extracted. Note that only colony intensities are discussed/reported in this study. Colony intensities were spatially corrected on each plate with the *SpatialBorderMedian()* function with *SpatialSmooth()* and *BorderMedian()* options. Growth curves were smoothed with *smoothdata()* using the *rlowess* option over a window of 48 timepoints (4 hr).

### Data analysis

Any colony with fewer than six data replicates in either untreated or UVR-treated conditions was removed. Data for colonies appearing &amp;gt;1x on the 6144-plate were regarded as extra replicates, resulting in analysis of 4294 unique strains. Due to overgrowth at later timepoints, the dataset was restricted to the first 40 hr of growth. Growth curves were normalized to a colony intensity of zero (total pixel intensity of colony). End-normalized curves were computed by normalizing each curve to its final colony intensity. Plate-specific reference curves were calculated as the median curve from all strains on a plate. Deviation profiles were calculated by comparing plate-specific reference curves to observed colony curves. LagVstall was computed from deviation profiles as the sum of distances between a given endpoint-normalized curve and the reference curve for that plate. Colony fitness was extracted as the final colony intensity of each colony on plates. LagVstall and colony fitness were *Z*-scored using MATLAB’s *normalize()* function with ‘robust’ settings, which normalizes to a median absolute deviation of one. Colony intensities or lagVstall were compared between UVR-treated and untreated conditions using *ttest2()*, and *q*-values were calculated using *mafdr()*, based on a previously defined method (Storey *et al.* 2002). Both *q*-values and uncorrected *p*-values are reported. Figures with shaded standard deviation around growth curves were generated with a modification of *stdshade()* (Musall 2010).

### GO term enrichment, other gene set enrichment

The dataset was filtered for the 95^th^ and 5^th^ percentiles of untreated lagVstall, resulting in 215 genes from each tail. These gene sets (Table S2) were tested for Gene Ontology (GO) term enrichment by hypergeometric test using MATLAB’s *hygedcdf()* as 1-*hygecdf(x-1,M,K,N)*, where *hygecdf()* calculates the probability of drawing up to *x* successes in *N* samples drawn without replacement from total population *K*, which contains *M* items with the desired characteristic. Significant GO terms were selected at an *q*-value cutoff of 0.05 (adjusted as described previously). Fold enrichment was calculated as the frequency of the term in the nominated strains divided by the frequency of the term in the overall dataset. Genes not present in the screen were not considered. Only enriched GO Biological Process terms are reported. GO Biological Process terms used for enrichment analysis were obtained from the GO Consortium (2020-01-01, http://doi:10.5281/zenodo.2529950).

DDR and mitochondrion-annotated gene sets were queried using YeastMine (Cherry *et al.* 2012; Balakrishnan *et al.* 2012). Specifically, the GO terms “mitochondrion” and “DNA damage response” (and children of these terms), as well as the phenotype “UV Resistance Reduced” were queried. Other gene sets were obtained from the indicated resources (Figure 3, Table S4). Hypergeometric tests and fold enrichment analysis were performed as described above. Genes not present in the screen were not considered. Three-way Venn diagrams were created with EulerAPE (Micallef and Rodgers 2014).

### YeastNet visualization

YeastNet v.3 (Kim *et al.* 2014) was downloaded and visualized in Cytoscape 3.8.0 (Shannon *et al.* 2003). The network was subsetted for genes nominated by either colony fitness or lagVstall. Note that these networks are slightly smaller than the full gene sets nominated in our screen due to YeastNet’s lack of ‘dubious ORFS’ (222/247 colony and 295/326 genes nominated by colony fitness and lagVstall, respectively). Edges with weights ≥ 1.5 were filtered. GO enrichment was performed and visualized on these subnetworks using BinGO (Maere *et al.* 2005). Alternatively, gene sets of interest were queried on YeastMine and visualized on the network.

### Data availability

The following items have been included as supplemental files in GSA Figshare: Descriptions of supplemental files (File_S1), 40-hour dataset (File_S3) including all pre-processed (spatially-corrected) and normalized replicate colony intensities; 24-hour restricted dataset (File_S4), scripts used for data processing (File_S5,6), and scripts required to reproduce figures presented in this paper (File_S7,8). The MATLAB Colony Toolkit Analyzer V2 software is available on GitHub (https://github.com/idekerlab/Matlab-Colony-Analyzer-Toolkit-v2.git). The following items are available upon request: raw image files in TIFF format, preliminary processed datasets, scripts used in image processing and plate normalization, and sequencing files/scripts for library strain identification. Supplemental material available at figshare: https://doi.org/10.25387/g3.12685667.

## Results

### High-throughput growth curve analysis with GEODE

**Figure 1:**
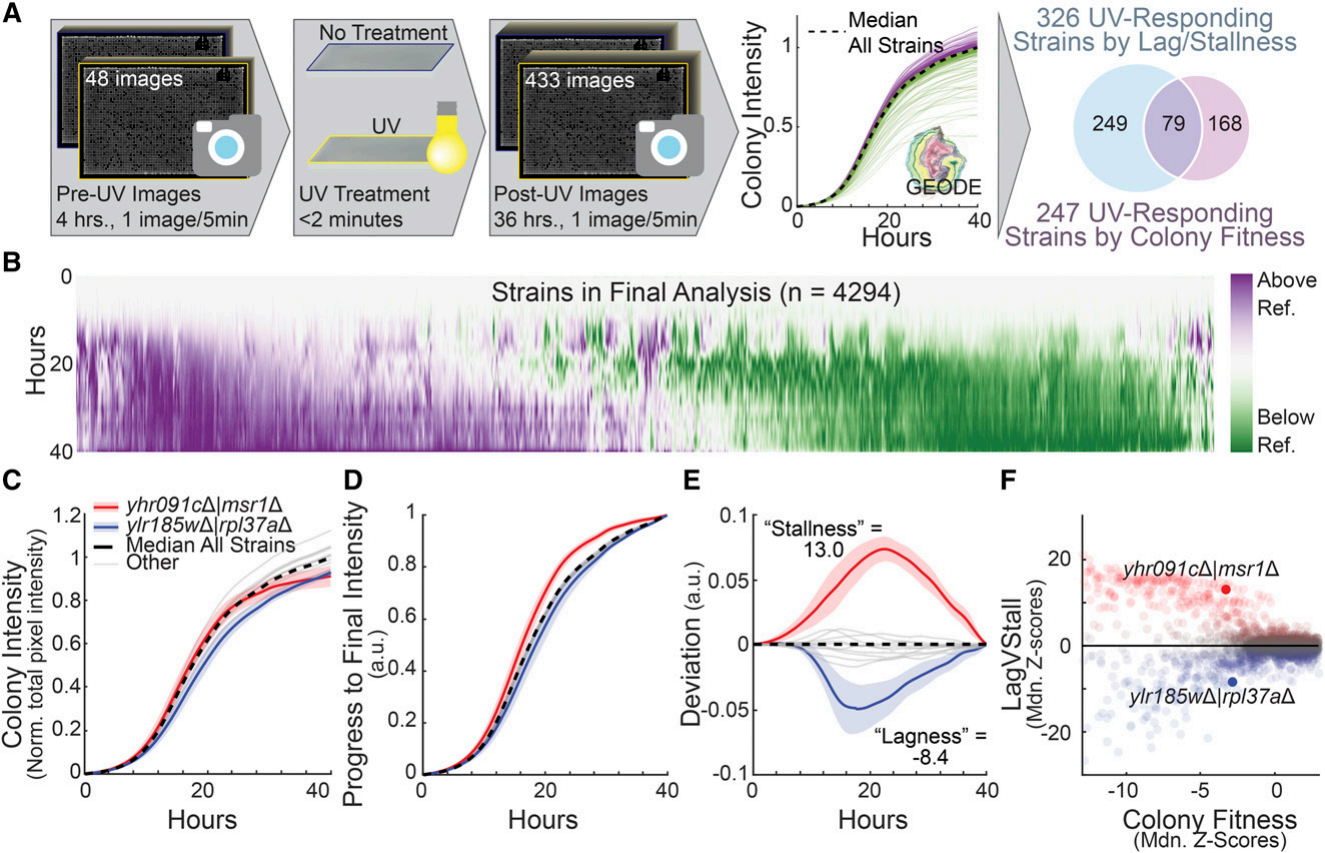
UVR Screen Pipeline. A) Schematic describing the UVR sensitivity screen. Plates were pinned and imaged for four hours at 5-minute intervals. Plates were then treated with UVR and imaging was resumed for 36 hr at 5-minute intervals. Growth curves were extracted and analyzed, resulting in the nomination of 326 genes by lagVstall (*q*-value cutoff = 0.05) and 247 strains by colony fitness (*q*-value cutoff = 0.05), with an overlap of 79 genes. B) Heatmap of growth curves obtained for all strains in untreated conditions. Purple and green coloring represent timepoints when a given curve existed above or below the median of all strains in the screen. C) Colony intensity (plate-normalized total pixel intensity) *vs.* time curves for a subset of ten strains and two strains of interest, *msr1*Δ *and* *rpl37a*Δ. Average curves are shown; shaded areas represent standard deviation. D) Endpoint-normalized growth curves for previously noted strains, reflecting progress to final colony intensity. E) Deviation profiles for previously noted strains. F) Median of non-treated replicate Z-scores for lagVstall *vs.* colony fitness (normalized pixel area).

We sought to establish a platform for the efficient capture and analysis of genome-wide dynamic growth curves. We achieved this platform by combining time-lapse imaging with an ultra-high-throughput 6144-colony array (Bean *et al.* 2014), which permits interrogation of an entire yeast gene deletion library on a single agar plate. We elected to screen non-essential strains using the homozygous diploid gene knockout library (Winzeler *et al.* 1999), which is less subject to the effects of secondary site mutations than the haploid library more typically used for genetic screens (Giaever and Nislow 2014). As each parental haploid strain involved in the creation of the diploid library had been generated via independent transformations, deleterious secondary site mutations are thus limited to two scenarios: the independent generation of the same mutation in both parental haploid strains, or deleterious haploinsufficient mutations created in a single parental haploid strain. To further improve screen quality, we verified the identity of all gene knockout loci via pooled barcode sequencing, updating strain annotations in 316 cases (Supplementary Methods, Fig S1A-D). The yeast library was robotically pinned in 6144-array format and imaged for 40 hr, (Figure 1A) with or without UVR treatment administered at 4 hr of growth. After spatial correction and selection for high-quality growth curves (Materials and Methods), we analyzed the growth of 4294 unique diploid knockout strains, encompassing, on average, 11 replicates per strain per treatment (Figure 1A, B).

We noted that many strains followed a similar growth trajectory, approximated by median population growth (dashed line, Figure 1C). We observed a diversity of growth trajectories about this curve (Figure 1B), raising the question of how to best identify, characterize and compare the significant differences. For example, consider the growth of strains deleted for the gene *MSR1*, encoding a nuclear-encoded mitochondrial tRNA synthetase, or *RPL37A*, encoding a 60s ribosomal subunit (Cherry *et al.* 2012). Both strains demonstrated decreased, yet similar, final colony intensities compared to the global population (Figure 1C). However, the two strains followed different growth trajectories in untreated conditions: *msr1*Δ tracked the population median trajectory for a short time, but then fell progressively behind the population, whereas *rpl37a*Δ grew slowly throughout the time course.

To standardize all growth curves for comparison, we normalized each curve to a final colony intensity of one, such that each normalized curve reflected progress of growth as a fraction of final colony intensity (Figure 1D). Post-normalization, we observed that many colonies now followed a similar trajectory (gray lines, Figure 1D) which was well-represented by the population median line (dashed line, Figure 1D). Conversely, the example strains were distinctly different: *msr1*Δ (red line, Figure 1D) lay distinctly above the median curve, while *rpl37a*Δ (blue line, Figure 1D) remained below the median curve.

**Figure 2:**
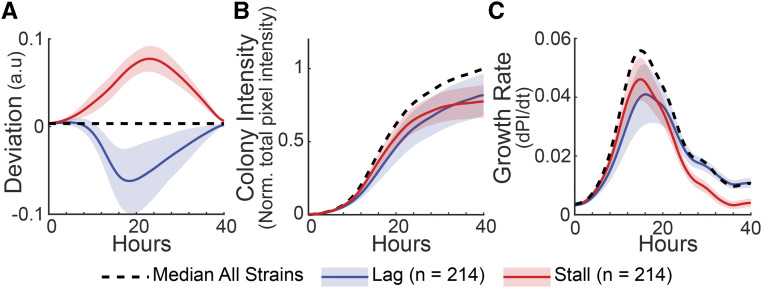
LagVstall Phenotypes. A) Deviation profiles for strains with extreme lagVstall. Average curves are shown; shaded area represents standard deviation of each group of 214 strains. B) Colony intensity (plate-normalized total pixel intensity) *vs.* time. C) Growth rate (dPI/dT; PI, pixel intensity) *vs.* time.

To quantitatively capture these differences, we calculated “deviation profiles” from the endpoint-normalized curves, reflecting the distance from each curve to the population median at any point in time (Figure 1E). We then calculated the integral of this curve, a growth-comprehensive metric which summarizes overall deviation of any particular growth curve from the population median. For reasons discussed below (Figure 2), we named this metric “lag,” when negative, and “stall,” when positive. Less fit colonies (determined by traditional endpoint analysis) exhibited more variable growth trajectories, and thus tended to have larger magnitudes of this metric, which we henceforth call lagVstall (wide range of lagVstall in Figure 1F for low colony fitness). Importantly, lagVstall distinguished the growth behaviors of *msr1*Δ and *rpl37a*Δ (Figure 1F).

### GEODE reveals dynamic growth phenotypes across mutant strains

We inspected the growth curves of strains with extreme lagVstall scores (5^th^, 95^th^ percentiles), which demonstrated strong deviation (Figure 2A). Stall strains (red line, Figure 2B) tended to closely follow the population trend for initial growth, but then stalled, falling progressively behind the population median (dashed line, Figure 2A). In contrast, lag strains (blue line, Figure 2B) tended to grow slowly for the duration of the experiment and stayed consistently below the population median. Similar trends were observed upon examination of growth rates: stall strains exhibited progressively slower growth rates compared to the population, while lag colonies started out with much slower growth rates that eventually matched the population during stationary growth (Figure 2C). We found that the lag gene set was enriched for gene functions involved in ribosome synthesis and translation (7/7 enriched Gene Ontology categories, Table S3), while the stall gene set was enriched for functions involved in respiration and mitochondria (8/9 enriched Gene Ontology categories, Table S3). Together, these results gave us confidence that lagVstall can translate diverse growth trajectories in a manner that integrates strain fitness and growth rates to inform biological function.

### Nomination of UVR-responders

**Figure 3:**
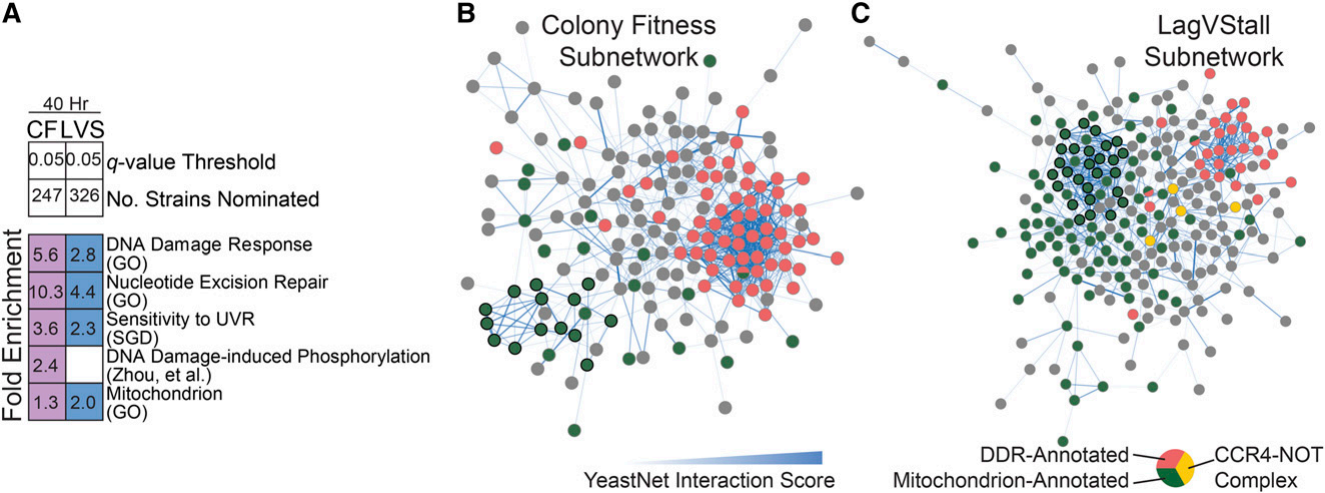
UVR-responsive Strains. A) Chart demonstrating results of gene set fold enrichments on strains nominated by colony fitness (CF) and lagVstall (LVS). Shading denotes significant result by hypergeometric test; cells with non-significant results have been left blank. Full results can be found in Table S4. B, C) CF and LVS-specific subnetworks of YeastNet V3, respectively, with edge weight thresholded to ≥ 1.5. Green denotes mitochondrial annotation; black border denotes annotation to mitochondrial ribosome. Pink denotes DDR-annotation. Yellow denotes components of the CCR4-NOT complex.

We next turned to the comparison of the UVR-treated (UVR) and untreated (UT) datasets. Initial inspection of the entire diploid gene deletion dataset demonstrated strong reproducibility with high correlation across replicates (⍴ = 0.97_UT:UT_; 0.92_UVR:UVR_), and even across treatments (⍴ = 0.92_UVR:UT_) (Figure S2A), indicating that most strains did not demonstrate a change in lagVstall due to treatment. We employed a *t*-test to compare untreated *vs.* UVR-treated lagVstall and colony fitness. This test nominated 494 genes whose knockout modulated the response to UVR; 168 strains were identified by colony fitness, 247 by lagVstall, and 79 by both metrics (*q*-value cutoff = 0.05, Table S5). We noted that 67 nominated strains were annotated to the DDR, representing 5.6 and 2.8-fold enrichments for sets of strains nominated by colony fitness and lagVstall, respectively. In addition, 70 nominated strains had previously been associated with UVR sensitivity (3.6- and 2.3-fold enrichment for colony fitness and lagVstall, respectively, Figure 3A and Table S4). We thus conclude that we have nominated a set of genes with functional relevance to the UVR response.

To further identify functional linkages among the nominated gene set, we visualized the significant results on YeastNet, an integrated gene-gene functional similarity network (Kim *et al.* 2014). One notable difference between the colony fitness and lagVstall sets was the differential abundance of DDR-annotated and mitochondrion-annotated genes. While colony fitness more robustly recovered DDR-annotated strains (62/247 strains, Figure 3B, Figure S3A, Table S4), lagVstall more robustly recovered mitochondrion-annotated strains (121/326 strains, Figure 3C, Figure S3B and Table S4). In the YeastNet subnetwork for colony fitness, DDR-annotated genes were tightly connected, while mitochondrial genes were more loosely connected, save for a dense cluster encoding components of the mitochondrial ribosome (green nodes with black border, Figure 3B). The lagVstall subnetwork demonstrated two densely connected clusters, corresponding to mitochondrial and DDR genes, respectively. The CCR4-Not complex was enriched in this network (yellow nodes, Figure 3C). We also identified components of autophagy and tRNA wobble uridine modification (Figure S3C).

**Figure 4:**
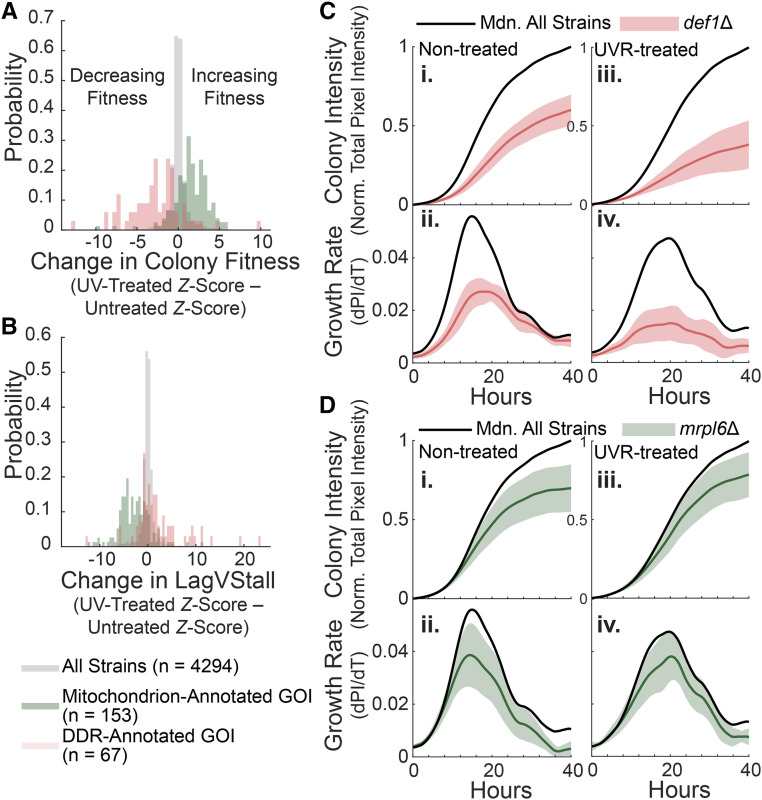
Characteristics of DDR and mitochondrial strains in response to UVR. A) Histogram of change in colony fitness (UVR - Untreated Z-scores). B) Histogram of change in lagVstall (UVR-Untreated *Z*-scores). C, D) Growth curves for *def1*Δ (red curves) and *mrpl6*Δ (green curves), respectively. Shaded area represents standard deviation; black line represents median curve for all strains in screen. i, Colony intensity (plate-normalized total pixel intensity) *vs.* time in untreated conditions; ii, Growth rate (dPI/dT) *vs.* time in untreated conditions; iii, Colony intensity (plate-normalized total pixel intensity) *vs.* time in UVR-treated conditions; iv, Growth rate (dPI/dT) *vs.* time in UVR-treated conditions.

Finally, we sought to understand differences in UVR response behavior for DDR *vs.* mitochondrial-deficient strains. Many DDR-deficient strains demonstrated reduced fitness (Figure 4A) and tended to shift toward a stall phenotype upon UVR treatment, either by increasing in stall phenotype severity or by overtly shifting from lag to stall (Figure 4B, Table S5). For example, we observed that disruption of *DEF1*, an RNAPII degradation factor associated with transcription-coupled NER, led to extremely slow growth in non-treated conditions that only matched population growth during stationary phase (Figure 4Ci, ii). UVR-treatment severely perturbed growth, preventing *def1*Δ from matching the population even during stationary phase (Figure 4Ciii, iv). In contrast, disruption of mitochondrion-annotated genes led to increased fitness (Figure 4A) and a switch from a strong stalling phenotype to a unique, less-severe stalling phenotype upon UVR treatment (Figure 4B, Table S5). For example, the strain *mrpl6*Δ, which is deficient in a component of the mitochondrial ribosome, fell progressively behind population growth in non-treated conditions (Figure 4Di, ii). However, UVR treatment reduced this difference such that *mrpl6*Δ did not fall behind as rapidly, resulting in a modest increase in relative fitness by the end of the screen (Figure 4Diii, iv).

## Discussion

In this study, we have applied GEODE, an ultra-high throughput dynamic growth analysis technique to study the UVR response. In addition to expected findings, such as involvement of DNA damage repair genes, we also highlight a role for mitochondria in this response.

### Screen design

We elected to screen the homozygous diploid knockout library. With two copies of each chromosome, phenotypes due to spurious mutations should be rare. One ongoing issue affecting such genome-wide screens, however, is the possibility of strain mixing or strain misidentification, as strains are stored in high-density arrays and handled almost exclusively with robotic tools. In an effort to minimize the impacts of mis-identified strains, we sequenced barcodes from our yeast homozygous diploid knockout library in its 96-well form. This resulted in identity correction for 316 strains. While it is possible that mixing or alterations could have been introduced at later screening stages, use of sequencing to verify strain identities was a crucial initial step toward maximizing data quality.

### Stall and lag growth phenotypes

Analysis of the dynamic growth data revealed two growth phenotypes: stall *vs.* lag. The lag trajectory is characterized by continuous poor growth. Strains demonstrating this phenotype were most enriched for ribosome synthesis functions. It can be inferred that these strains are deficient in fully-functional ribosomes and may thus be translation-incompetent (Steffen *et al.* 2008, 2012), potentially explaining the depressed growth trajectories we and others have observed (Warringer *et al.* 2003; Steffen *et al.* 2008, 2012).

The stall trajectory is characterized by a period of growth that resembles the population, after which the colony of interest stalls, falling progressively behind. Strains exhibiting this phenotype were most enriched for mitochondrial functions. Our use of glucose-containing medium may explain enrichment for these functions. When present, glucose promotes ATP generation by fermentation; enzymes required for metabolism of other carbon sources only appear when glucose becomes limiting (Gancedo 1998; Merz and Westermann 2009). Thus, growth defects for respiration-deficient strains are only observed when glucose is limiting and a switch to aerobic respiration is required.

### UVR-deviant strains

In our application to the UVR response, we nominated 494 UVR-responding genes at an *q*-value cutoff of 0.05. 67 of these strains have a previously identified role in the DDR, known sensitivity to UVR, or both; 301 have known or predicted human orthologs, and therefore may be functionally relevant outside of *Saccharomyces*. Additionally, we noted that knockout strains identified by lagVstall trended strongly toward resistance, while strains nominated by colony fitness trended toward sensitivity, highlighting the need to examine both static (colony fitness) and dynamic (lagVstall) metrics to gain a full picture of the UVR-induced response.

### Phenotypes of DDR-annotated strains

A subset of DDR-annotated strains tended to exhibit lag phenotypes in non-treated conditions. DDR-deficient strains are known to be afflicted by higher-than-usual basal mutation rates, aneuploidies, and chromosomal rearrangements (Evert *et al.* 2004; Serero *et al.* 2014); consequences of increased basal mutation include abnormal cell growth, morphology, and increased DNA content (Evert *et al.* 2004), all of which could conceivably contribute to a lag phenotype. Notably, UVR treatment caused a shift toward stalled growth for some DDR-deficient strains, such as *def1*Δ. The overall impact of UVR treatment is to slow growth until cells repair DNA damage. While most strains recovered rapidly from UVR treatment, DDR-deficient strains, such as *def1*Δ, were likely unable to repair damage. The impediment to growth endured into the stationary growth phase, thus producing a stall phenotype in some of these strains.

### Mitochondrial-annotated UVR-deviant strains

Mitochondria produce ATP and play important roles in amino acid, nucleotide, and Fe-S cluster cofactor metabolism (Malina *et al.* 2018); they are additionally a significant source of intracellular reactive oxygen species. While it is known that nuclear-mitochondrial cross-talk mediates coordination between the cell and its energetic factory (Saki and Prakash 2017), the exact relationship between mitochondria and DNA damage remains unresolved. Some studies report transcriptional repression (Gasch *et al.* 2001; Jaehnig *et al.* 2013) or inhibition of respiratory activity (Kitanovic *et al.* 2009) in response to DNA damage, while other studies report a protective role for respiration in response to DNA damage (Sung *et al.* 2010; Bu *et al.* 2019). Uncertainties regarding the role of mitochondria extend further to tumorigenesis, where mitochondrial abnormalities have long been observed.

We were surprised to find that many strains deficient in genes annotated to mitochondria were relatively resistant to UVR treatment. It is possible that slowed growth due to UVR treatment was associated with slower glucose depletion and thus prolonged anaerobic growth. However, prolonged anaerobic growth would equally benefit all strains, since glucose inhibits respiration. Instead, our results would seem to support a role for mitochondrial impairment in improved recovery from UVR, as evidenced by weakening of stall growth phenotype for strains such as *mrpl6*Δ. One possible explanation is that an increased basal level of nuclear DNA damage resulting from mitochondrial impairment (Rasmussen *et al.* 2003) ‘primes’ cells to respond to subsequent induced DNA damage. If so, the protective effects of mitochondrial impairment may be specific to the damaging agent; differential resistance of respiration-deficient strains to H_2_O_2_ and 4NQO has indeed previously been reported (Rasmussen *et al.* 2003). Further supporting the possibility of damage type specificity, 47 knockout strains whose gene products localize to the mitochondrion were previously identified in another screen for UVR sensitivity, but not 4NQO sensitivity (Begley *et al.* 2004). Further research will be required to determine the mechanism by which mitochondrial impairment may specifically influence resistance to UVR-induced DNA damage.

### Other UVR-deviant groups

We identified four components of the CCR4-NOT complex, which regulates nucleotide production in response to replication stress and DNA damage via induction of ribonucleotide reductase genes following treatment (Mulder *et al.* 2005). Consistent with previous results, three knockout strains (*ccr4*Δ, *mot2*Δ, *and* *pop2*Δ*)* demonstrated sensitivity to UVR and other damaging treatments, and one strain (*caf16*Δ) did not. It is notable that this strain was identified on the basis of lagVstall in our screen, and not strain fitness, possibly indicating a transient UVR-associated phenotype that has yet to be investigated.

We additionally noted autophagy and tRNA wobble uridine modification components on the basis of lagVstall but not colony fitness. It is well accepted that autophagy is induced in response to DNA damage and plays roles in both repair of damage as well as cell death resulting from DNA damage (Eliopoulos *et al.* 2016). Likewise, modification of the wobble position on tRNAs has been shown to be important in the production of selenoproteins, which are involved in the detection of reactive oxygen species (Endres *et al.* 2015). Notably, inspection of corresponding growth curves revealed few obvious changes in growth pattern or strain fitness.
